# Validation of Taijiquan (Tai Chi) Exercise Commitment Scale for Chinese Elderly (TECS-CE)

**DOI:** 10.3389/fpsyg.2026.1889773

**Published:** 2026-09-16

**Authors:** Sun Xianghao, Kritchapol Arsapakdee, Chairat Choosakul

**Affiliations:** 1 Faculty of Physical Education, Henan Polytechnic University, Jiaozuo, China; 2 Department of Health and Sport Science, Faculty of Education, Mahasarakham University, Maha Sarakham, Thailand

**Keywords:** Exercise Commitment Scale, older adults, physical activity, psychometric validation, Tai Chi, Taijiquan

## Abstract

**Background:**

Taijiquan, also widely known as Tai Chi, is a traditional Chinese mind–body exercise that has been associated with physical and psychological health benefits among older adults. Despite these benefits, sustained participation may be influenced by psychological commitment and contextual determinants. A population-specific measure is therefore needed to assess commitment to Taijiquan exercise among older adults. This study aimed to examine the psychometric properties of the Taijiquan Exercise Commitment Scale for Chinese Elderly (TECS-CE).

**Methods:**

A cross-sectional survey was conducted among 453 older adults aged 60–75 years who practiced Taijiquan in Jiaozuo City, Henan Province, China. The participants were divided into an exploratory factor analysis (EFA) sample (*n* = 203) and a confirmatory factor analysis (CFA) sample (*n* = 250). Thirty participants completed the scale twice with a 3-week interval to examine test–retest reliability. Content validity was evaluated by five experts using the Index of Item–Objective Congruence (IOC). Construct validity was examined using EFA and CFA, while criterion-related validity was examined through correlations and regression analyses with self-reported Taijiquan exercise behavior.

**Results:**

The final TECS-CE contained 33 items representing two commitment dimensions—Want to Commit and Have to Commit—and six determinants: Satisfaction, Social Constraints, Involvement Alternatives, Personal Investment, Social Support, and Involvement Opportunities. The two commitment dimensions explained 65.005% of the variance, while the six determinants explained 75.378%. CFA indicated acceptable model fit for the reported models; for the structural model, χ^2^/df = 1.371, RMSEA = 0.039, RMR = 0.072, AGFI = 0.882, GFI = 0.903, NNFI = 0.984, and CFI = 0.986. Composite reliability ranged from 0.91 to 0.94 and AVE ranged from 0.68 to 0.71. Cronbach’s alpha coefficients ranged from 0.932 to 0.984, and test–retest coefficients ranged from 0.824 to 0.938. The TECS-CE dimensions and determinants were significantly associated with self-reported exercise behavior.

**Conclusion:**

The findings provide evidence that the TECS-CE has acceptable content, construct, criterion-related, and reliability evidence for assessing Taijiquan exercise commitment among the older adults included in this study. Because the study was cross-sectional and used convenience sampling and self-reported exercise behavior, longitudinal and multisite studies are needed to establish predictive validity and broader generalizability.

## 1 Introduction

Population aging has become a major global public health challenge, requiring effective strategies to improve the health and quality of life of older adults. The World Health Organization emphasized the importance of maintaining healthy behaviors throughout the lifespan, particularly through regular physical activity and strength training to preserve muscle mass and functional capacity among elderly populations (World Health Organization, 2018). However, despite extensive public health efforts, global physical activity levels remain insufficient and continue to show limited improvement (Lightfoot et al., 2018). This issue is especially significant in China, where the aging population has increased rapidly in recent decades.

Taijiquan, a traditional Chinese exercise rooted in Chinese medicine and philosophy, has been widely recognized as an effective form of physical activity for promoting health and preventing disease among older adults (Li, 2016). Previous studies have demonstrated that Taijiquan provides multiple physical and psychological benefits, including improved balance, enhanced mobility, reduced fall risk, and better overall well-being (Guo et al., 2016). In particular, systematic reviews and meta-analyses have confirmed the effectiveness of Taijiquan in fall prevention among elderly populations (Lomas-Vega et al., 2017). Consequently, the Chinese government incorporated Taijiquan promotion into the “Healthy China 2030” initiative as an important national strategy for health promotion and active aging (Official Website of the Chinese Government, 2024). International organizations such as the World Health Organization and the Centers for Disease Control and Prevention have also integrated Taijiquan into community-based exercise and fall-prevention programs for older adults (Kalache et al., 2007). Furthermore, the U.S. Centers for Disease Control and Prevention identified Taijiquan as one of the most effective exercise interventions for reducing fall risk among community-dwelling elderly individuals (Stevens and Burns, 2010). Similarly, the U.S. Administration for Community Living endorsed Taijiquan as an evidence-based exercise program for fall prevention and healthy aging (Li et al., 2005).

Despite these recognized benefits and policy endorsements, participation and long-term adherence to Taijiquan exercise among older adults remain inconsistent. Many elderly individuals discontinue participation due to barriers such as dissatisfaction with teaching methods, lack of motivation, reduced enjoyment, and limited social support (Matsuura, 2019). Previous studies have suggested that interventions should be adapted to better correspond with the needs, preferences, and lifestyles of elderly practitioners in order to improve sustained participation and exercise adherence (Stevens et al., 2014).

Exercise commitment has been identified as an important psychological construct associated with exercise adherence and long-term participation behaviors. Commitment refers to an individual’s psychological determination, obligation, or desire to continue participation in a particular activity (Corbin et al., 1987). To better understand factors influencing sport participation, Scanlan et al. developed the Sport Commitment Model (SCM), which explains the psychological processes associated with continued participation in sport and exercise contexts (Choosakul et al., 2009; Scanlan et al., 1993). Subsequently, Wilson et al. developed the Exercise Commitment Scale (ECS) based on the SCM framework and demonstrated its effectiveness in predicting exercise behavior among participants (Wilson et al., 2004). Building upon this work, Qiu et al. adapted and validated the ECS among Chinese college students, confirming its applicability across different cultural and demographic contexts (Qiu et al., 2012).

Nevertheless, no validated instrument currently exists to specifically assess Taijiquan exercise commitment among elderly populations. This limitation represents a significant research gap, particularly because exercise commitment has been shown to strongly influence exercise adherence and participation behaviors (Chen et al., 2005). Furthermore, previous research has suggested that the determinants of commitment within the Sport Commitment Model may vary across different populations and activity contexts, thereby requiring additional validation when applied to elderly individuals and culturally specific physical activities such as Taijiquan (Wilson et al., 2004). Given the unique cultural characteristics of Taijiquan and the distinct physical, psychological, and social needs of older adults, the development of a specialized instrument for measuring Taijiquan exercise commitment among elderly populations is both necessary and important.

Although the Exercise Commitment Scale (ECS) developed by Wilson et al. has been widely applied in exercise settings, the instrument was originally designed for general exercise participants and has primarily been validated among younger populations (Wilson et al., 2004). Likewise, the Chinese adaptation by Qiu et al. focused on college students. Consequently, the psychometric properties and conceptual structure of the ECS have not been established for Chinese older adults who regularly practice Taijiquan. Because exercise commitment may be influenced by age, cultural context, and the characteristics of a specific physical activity, applying an existing instrument without population-specific validation may result in an inaccurate assessment of commitment among older Taijiquan practitioners (Qiu et al., 2012).

Furthermore, Taijiquan differs from many conventional forms of exercise because it integrates slow, controlled movements with traditional Chinese philosophy, mindfulness, postural control, body awareness, and social interaction. These characteristics may shape the determinants of exercise commitment in ways that differ from those observed in conventional exercise settings. Importantly, the physical characteristics of Taijiquan may also be particularly relevant to older adults because its slow and controlled movements can support postural stability, functional movement, and mind–body integration, while potentially providing neuro-musculoskeletal and somatovisceral benefits associated with healthy aging (Lee and Chu, 2023). Such characteristics connect psychological commitment to the physical safety, functional capacity, and biomechanical feasibility of sustained participation. Therefore, understanding exercise commitment in older Taijiquan practitioners requires an instrument that captures not only psychological and behavioral dimensions but also the distinctive physical and mind–body characteristics of this traditional exercise.

Accordingly, it is necessary to develop and validate a measurement instrument specifically designed for Chinese older adults who practice Taijiquan. A valid and reliable instrument will enable researchers, health professionals, and policymakers to accurately assess exercise commitment, identify factors associated with long-term participation, and develop evidence-based interventions that support sustained Taijiquan practice and healthy aging. Therefore, the present study examined the psychometric properties of the Taijiquan Exercise Commitment Scale for Chinese Elderly (TECS-CE), focusing on content validity, factor structure, convergent and discriminant validity, criterion-related validity, internal consistency, and test–retest reliability. The findings are intended to provide a population- and activity-specific instrument for assessing Taijiquan exercise commitment among older adults.

## 2 Materials and methods

### 2.1 Research design

This study employed a quantitative, cross-sectional survey design to examine the psychometric properties of the Taijiquan Exercise Commitment Scale for Chinese Elderly (TECS-CE), with particular emphasis on its validity and reliability (Cronbach, 1951; West, 1995). A cross-sectional survey design was considered appropriate because it enabled the systematic collection of quantitative data from a relatively large group of older adults within a defined period.

Convenience sampling, a non-probability sampling technique, was used to recruit participants from accessible Taijiquan exercise groups in Jiaozuo City, Henan Province, China. This approach is commonly used in exploratory and social science research when participant accessibility and feasibility of data collection are important considerations (Turner, 2020). The use of convenience sampling was therefore appropriate for accessing older adults who regularly practiced Taijiquan and met the predetermined eligibility criteria.

The study focused on the empirical validation of the TECS-CE through a series of psychometric analyses. The factor structure was initially examined using exploratory factor analysis (EFA) and subsequently tested using confirmatory factor analysis (CFA). Additional analyses were conducted to evaluate convergent validity, discriminant validity, criterion-related validity, internal consistency, and test–retest reliability. The cross-sectional design permitted the examination of associations among the measured constructs but did not allow temporal or causal relationships to be established.

### 2.2 Participants

#### 2.2.1 Sampling size

The sample size was determined according to established recommendations for psychometric validation studies. Methodological guidelines generally recommend approximately 5–10 participants per item for factor analysis, with at least 200 participants for confirmatory factor analysis (CFA), and a total sample of approximately 300–500 participants for scale development and validation (Hair et al., 2019; Kline, 2016; Worthington and Whittaker, 2006). As the initial Taijiquan Exercise Commitment Scale for Chinese Elderly (TECS-CE) consisted of 34 items, a minimum sample of approximately 340 participants was considered appropriate. Accordingly, a total of 453 older adults who regularly practiced Taijiquan were recruited, exceeding the recommended sample size and providing sufficient observations for both exploratory factor analysis (EFA) and CFA.

Prior to the factor analyses, the initial item pool underwent expert consultation and preliminary pre-testing to establish content validity and assess the clarity and comprehensibility of the items. The expert panel evaluated the relevance, clarity, and appropriateness of each item for older adults who practiced Taijiquan. Content validity was assessed using the item-level Content Validity Index (I-CVI), with items meeting the predetermined criterion retained for subsequent analysis. Following expert review, the revised questionnaire was pre-tested with a separate group of older Taijiquan practitioners to identify ambiguous wording, difficulties in comprehension, and problems with questionnaire administration before the formal factor analyses. The final questionnaire used a five-point Likert scale ranging from 1 (strongly disagree) to 5 (strongly agree) (see Supplementary Appendix). Participants were instructed to select the response that best represented their actual feelings, attitudes, or experiences regarding Taijiquan exercise commitment.

The total sample of 453 participants comprised 246 males (54.30%) and 207 females (45.70%). To provide independent evidence for the factor structure, the sample was divided into two subsamples: Sample 1 (*n* = 203) was used for EFA, and Sample 2 (*n* = 250) was used for CFA. This approach reduced the risk of capitalizing on chance and allowed the factor structure identified through EFA to be independently evaluated through CFA. In addition, Thirty participants completed the TECS-CE twice, with a 3-week interval between administrations, to assess test–retest reliability.

#### 2.2.2 Sampling procedure

Participants were recruited between January 10 and March 20, 2022, from organized Taijiquan exercise groups in China using a convenience sampling approach. Permission to conduct data collection was obtained from the leaders of local Taijiquan organizations before recruitment. Trained research assistants administered the questionnaires during scheduled Taijiquan exercise sessions.

Before participation, all respondents received a detailed explanation of the study objectives, procedures, and voluntary nature of participation. Participants were informed that their responses would be kept confidential and used solely for research purposes. Written informed consent was obtained from all participants before data collection. The questionnaire required approximately 5–10 min to complete.

#### 2.2.3 Participant eligibility criteria

Participants were eligible if they were aged 60–75 years, were physically capable of participating in regular Taijiquan exercise, could complete the questionnaire independently, agreed to complete all study procedures, and had at least 3 years of Taijiquan exercise experience. These criteria were established to ensure that participants had sufficient experience with Taijiquan to provide meaningful responses regarding their exercise commitment.

A total of 453 older adults participated in the study. The participants were aged 60–75 years and had at least 3 years of Taijiquan exercise experience. The sample comprised 246 males (54.30%) and 207 females (45.70%).

The total sample was divided into two independent subsamples for psychometric analysis: Sample 1 (*n* = 203) for EFA and Sample 2 (*n* = 250) for CFA. Thirty additional participants completed the questionnaire twice with a 3-week interval for the assessment of test–retest reliability. Although this sampling strategy facilitated the recruitment of experienced older Taijiquan practitioners, the use of convenience sampling within a limited geographical setting and the requirement of at least 3 years of Taijiquan experience may restrict the generalizability of the findings to novice practitioners, individuals with lower adherence, rural populations, institutionalized older adults, or other older-adult populations with different demographic and exercise characteristics.

### 2.3 Measurement instruments and validity

#### 2.3.1 Exercise Commitment Scale

The Taijiquan Exercise Commitment Scale for Chinese Elderly (TECS-CE) was adapted from the Exercise Commitment Scale (ECS) developed by Wilson et al. (2004) and its Chinese adaptation reported by Qiu et al. (2012). Wilson et al. (2004) developed the ECS based on the Sport Commitment Model (SCM) to examine exercise commitment and its relationship with exercise behavior. Subsequently, Qiu et al. (2012) adapted and examined the ECS among Chinese college students. However, the existing ECS and its Chinese adaptation were developed for general exercise or younger populations and were not specifically designed for older adults who regularly practice Taijiquan. Therefore, the present study adapted the ECS and its Chinese version by modifying the wording and exercise context of the items to reflect the experiences of older adults practicing Taijiquan, while retaining the underlying conceptual structure of the original instrument (Qiu et al., 2012; Wilson et al., 2004).

The translation and adaptation procedure followed the forward–backward translation approach reported by Qiu et al. (2012). In their Chinese adaptation of the ECS, the original English version was first translated into Chinese by a bilingual translator proficient in both English and Chinese. The Chinese version was then independently back-translated into English by another bilingual translator. Subsequently, an English-language lecturer and a sport psychology researcher reviewed the forward- and back-translated versions to identify discrepancies and ensure linguistic and conceptual consistency. The final version was established as the Chinese version of the ECS (Qiu et al., 2012).

Qiu et al. (2012) also examined language equivalence using a bilingual testing procedure before the Chinese ECS was used for subsequent psychometric analyses. Sixty university students participated in the language equivalence test and were assigned to four groups according to the language used at the pretest and posttest: English–English, English–Chinese, Chinese–English, and Chinese–Chinese. The two testing sessions were separated by 3 weeks. Most items demonstrated language equivalence coefficients above 0.90. Two items with lower coefficients were retranslated and revised, after which their coefficients increased to 0.76 and 0.84, respectively. These items were subsequently retained for further analyses. This procedure provided a methodological reference for the translation and linguistic adaptation of the TECS-CE.

Based on this framework, the present study developed the initial TECS-CE by adapting the wording and exercise context of the ECS items to reflect the experiences of Chinese older adults who regularly practiced Taijiquan. The initial questionnaire comprised 34 items representing two major commitment dimensions and six commitment determinants. The adapted items were reviewed for linguistic clarity, conceptual appropriateness, and consistency with the Taijiquan exercise context before expert evaluation.

Five experts independently evaluated the initial items using the Index of Item–Objective Congruence (IOC). The expert panel was selected based on their academic and professional expertise in exercise science, Taijiquan, physical education, sport psychology, and physical activity among older adults. Collectively, the experts had relevant academic and professional experience in exercise and physical activity research, Taijiquan practice or instruction, and the development and evaluation of measurement instruments. Their expertise also covered the assessment of conceptual appropriateness, clarity, and applicability of questionnaire items for older adult populations.

Following the first round of evaluation, items with IOC values below 0.60 were revised, and the revised items were subsequently subjected to a second round of expert evaluation. The final IOC results indicated an average value of 0.80 for 10 items and 1.00 for the remaining items, supporting the content relevance and congruence of the adapted items.

The TECS-CE was administered using a five-point Likert scale ranging from 1 (strongly disagree) to 5 (strongly agree). Participants were instructed to select the response that best represented their actual feelings, attitudes, or experiences regarding their commitment to Taijiquan exercise.

#### 2.3.2 Exercise Behavior Index

The Exercise Behavior Index was used as an external behavioral criterion to examine the criterion-related validity of the TECS-CE. The index was developed based on three indicators of exercise behavior—exercise frequency, exercise duration, and perceived exercise intensity—and one indicator of exercise persistence (Qiu et al., 2012).

Exercise behavior was assessed using four self-report questions. First, exercise frequency was assessed by asking, “How many times do you practice Taijiquan every week?” with four response options: (A) 1–3 times, (B) 3–5 times, (C) 5–7 times, and (D) more than 7 times. Second, exercise duration was assessed by asking, “For how long do you practice Taijiquan each time?” with four response options: (A) approximately 30 min, (B) approximately 1 h, (C) 1–1.5 h, and (D) more than 1.5 h. Third, perceived exercise intensity was assessed by asking, “What changes happen to your body after every Taijiquan exercise?” with five response options: (A) no noticeable change, (B) mild warmth or fever, (C) mild sweating, (D) sweating, and (E) heavy sweating. Fourth, exercise persistence was assessed by asking, “For how long have you been practicing Taijiquan?” with four response options: (A) 3 months to 1 year, (B) 1–3 years, (C) 3–5 years, and (D) more than 5 years.

The four indicators were used to characterize participants’ regularity, duration, perceived intensity, and persistence of Taijiquan participation. Associations between the TECS-CE scores and the Exercise Behavior Index were examined as evidence of criterion-related validity, with stronger exercise commitment expected to be associated with more frequent, sustained, and regular Taijiquan participation.

Because the Exercise Behavior Index relies on self-reported exercise behavior, the responses may be subject to recall and social-desirability bias. In particular, perceived exercise intensity was assessed partly through bodily changes and sweating, which may vary according to individual physiological characteristics and environmental conditions. Future studies should consider incorporating objective measures, such as accelerometers or heart-rate monitoring, to provide more robust behavioral criteria for evaluating the criterion-related validity of the TECS-CE.

### 2.4 Data analysis

All statistical analyses were performed using IBM SPSS Statistics 32.0 and IBM SPSS Amos 32.0. Before conducting the psychometric analyses, the dataset was screened for completeness and distributional characteristics. Descriptive statistics, including means, standard deviations, skewness, and kurtosis, were calculated for the observed variables. The dataset was also examined for incomplete questionnaires and missing responses. The number of incomplete cases and the procedure used to address missing data were documented according to the procedures applied to the original dataset.

Exploratory factor analysis (EFA) was conducted to examine the underlying factor structure of the TECS-CE. EFA was performed separately for the commitment dimensions and commitment determinants. The suitability of the data for factor analysis was evaluated using the Kaiser–Meyer–Olkin (KMO) measure of sampling adequacy and Bartlett’s test of sphericity. The factor structure was examined using principal component analysis (PCA) with oblique rotation. Oblique rotation was selected because the extracted factors were theoretically expected to be related, and the observed inter-factor correlations indicated moderate associations among the factors. Factor retention was determined based on the eigenvalue criterion and inspection of the scree plot. Factor loadings and the conceptual interpretability of the extracted factors were also considered when evaluating the final factor structure. Internal consistency reliability was assessed using Cronbach’s coefficient alpha (α) (Cronbach, 1951).

Following EFA, confirmatory factor analysis (CFA) was conducted using an independent calibration sample to cross-validate the factor structure identified through the exploratory analysis. CFA procedures were used to examine the viability of the commitment determinant and dimension structures derived from the EFA (Qiu et al., 2012; Scanlan et al., 1993). Model fit was evaluated using multiple complementary indices, including the chi-square statistic divided by degrees of freedom (χ^2^/df), root mean square error of approximation (RMSEA), Root Mean Square Residual (RMR) goodness-of-fit index (GFI), adjusted goodness-of-fit index (AGFI), non-normed fit index (NNFI), and comparative fit index (CFI). The CFI was used to evaluate the comparative fit of the hypothesized model relative to the null model (Cronbach, 1951), whereas the GFI was used as an index of the overall goodness of model fit (Mulaik et al., 1989). The χ^2^/df ratio was also considered as an index of model fit (Kelloway, 1998). Standardized factor loadings were examined to assess the contribution of individual items to their respective latent constructs.

Convergent validity was evaluated using standardized factor loadings, composite reliability (CR), and average variance extracted (AVE). Adequate convergent validity was assessed based on the magnitude and statistical significance of factor loadings, together with the CR and AVE values. Discriminant validity was examined using the Fornell–Larcker criterion by comparing the square root of the AVE for each construct with its correlations with the other constructs.

Internal consistency reliability was assessed using Cronbach’s coefficient alpha (α) and composite reliability (CR). Test–retest reliability was evaluated using data from participants who completed the TECS-CE on two occasions separated by a 3-week interval. The stability of scores across the two administrations was examined using the appropriate reliability coefficient.

Criterion-related validity was examined by assessing the associations between TECS-CE scores and self-reported Taijiquan exercise behavior. The exercise behavior indicators included exercise frequency, duration of each exercise session, perceived exercise intensity, and exercise persistence. Pearson correlation analysis was used to examine the relationships between exercise commitment and the exercise behavior indicators. Regression analysis was subsequently conducted to examine the statistical associations between exercise commitment and exercise behavior. Given the cross-sectional design, regression coefficients were interpreted as statistical associations rather than evidence of temporal prediction or causal effects.

Statistical significance was evaluated using a two-tailed significance level of *p* < 0.05. All analyses were interpreted collectively to evaluate the validity, reliability, and applicability of the TECS-CE for assessing exercise commitment among older adults who practice Taijiquan.

### 2.5 Ethics statement

Ethical approval for this study was obtained from the Ethics Review Committee of Mahasarakham University, Thailand, and the Medical Research Ethics Review Committee of Henan Polytechnic University (No. 2021-36, April 4, 2021). Participation was voluntary, and all participants provided written informed consent prior to data collection.

## 3 Results

### 3.1 The validation and calibration samples

Before starting the analysis, the researcher analyzed the statistical values (means, standard deviations, skewness, and kurtosis) necessary for use in the structural equation modeling testing using SPSS 32.0. Table 1 presents the descriptive statistics for all the variables observed by group status to validate the samples and calibration samples in this study. The means and standard deviations, from 203 of the validation samples were 1.916–4.690 and 0.4743–0.8646, respectively. Means and standard deviations for the number of 250 of the calibration samples were 1.916–4.690 and 0.4743–0.8646 respectively. The skewness and kurtosis values of all the observed variables in both the validation and calibration samples were lower than 3.75, which is acceptable for the assumption of multivariate normality (Tabachnick et al., 2013). Therefore, these data were deemed suitable for analysis, as shown in Table 1.

**TABLE 1:** Presents descriptive statistics for all the observed variables for the validation and calibration samples elderly Taijiquan exercisers.

Items	Validation samples (*N* = 203)				Calibration samples (*N* = 250)			
	*M*	SD	Skewness	Kurtosis	*M*	SD	Skewness	Kurtosis
WC1	4.448	0.5725	−0.435	−0.755	3.925	1.0884	−1.164	0.918
WC2	4.291	0.5622	−0.047	−0.538	3.797	1.0573	−1.042	0.783
WC3	4.207	0.5942	−0.097	−0.399	3.795	1.0828	−1.066	0.735
WC4	4.044	0.6395	−0.038	−0.533	3.700	1.0318	−0.927	0.627
WC5	4.374	0.5520	−0.112	−0.853	3.863	1.0824	−1.051	0.636
WC6	4.089	0.5904	−0.021	−0.149	3.728	1.0701	−1.009	0.638
HC1	4.084	0.7021	−0.378	−0.074	3.731	1.0424	−0.821	0.332
HC2	4.330	0.6558	−0.573	−0.165	3.837	1.1150	−1.011	0.513
HC3	4.187	0.6483	−0.202	−0.673	3.788	1.0429	−0.966	0.657
Sat1	4.404	0.6006	−0.454	−0.652	3.843	1.0807	−0.974	0.521
Sat2	4.404	0.6088	−0.490	−0.632	3.850	1.0928	−0.966	0.401
Sat3	4.483	0.5999	−0.697	−0.466	3.894	1.1337	−1.035	0.394
SC1	2.719	0.8646	0.299	0.184	2.660	1.0323	0.451	−0.190
SC2	2.433	0.8321	0.031	−0.035	2.492	1.0275	0.439	−0.058
SC3	1.916	0.7885	0.211	−1.189	2.260	1.0759	0.698	0.017
SC4	2.433	0.8321	−0.021	−0.307	2.481	1.0613	0.529	−0.082
IA1	2.394	0.6233	0.223	−0.107	2.499	0.9992	0.625	0.243
IA2	2.409	0.6489	0.563	0.799	2.510	0.9880	0.774	0.412
IA3	2.335	0.6419	0.354	0.149	2.450	1.0328	0.733	0.216
IA4	2.340	0.6583	0.241	0.672	2.450	0.9935	0.648	0.297
IA5	2.138	0.7111	0.211	−0.135	2.353	1.0126	0.687	0.205
PI1	4.158	0.6252	−0.125	−0.509	3.715	1.0687	−0.898	0.363
PI2	4.172	0.6252	−0.141	−0.526	3.735	1.1173	−0.955	0.378
PI3	4.187	0.6406	−0.188	−0.630	3.744	1.1213	−0.901	0.218
PI4	3.557	0.8148	−0.184	−0.446	3.035	1.0082	−0.240	−0.459
SS1	4.606	0.5380	−0.913	−0.259	3.949	1.1462	−1.105	0.474
SS2	4.690	0.4743	−0.966	−0.702	3.980	1.1548	−1.121	0.504
SS3	4.685	0.4763	−0.939	−0.759	3.971	1.1661	−1.172	0.603
IO1	4.227	0.6117	−0.169	−0.524	3.804	1.0596	−0.991	0.675
IO2	4.212	0.6129	−0.155	−0.508	3.819	1.0339	−0.923	0.532
IO3	4.389	0.5370	−0.032	−1.026	3.857	1.0784	−1.062	0.669
IO4	4.429	0.5157	0.071	−1.487	3.912	1.0648	−1.137	0.869
IO5	4.458	0.4995	0.169	−1.991	3.885	1.0664	−1.023	0.612
IO6	4.296	0.5552	−0.013	−0.557	3.821	1.0565	−0.995	0.687

WC, want to commit; HC, have to commit; IA, involvement alternatives; IO, involvement opportunities; PI, personal investment; SC, social constraints; Sat, satisfaction; SS, social support.

### 3.2 Exercise behavior

From the scores of exercise time, exercise frequency, exercise intensity, and exercise persistence, the average time for the elderly to participate in Taijiquan exercise activities was over an hour each time, the average number of Taijiquan exercise activities per week was over three times, and the intensity of each Taijiquan exercise caused slight to moderate sweating. Most elderly people have developed a habit of Taijiquan exercise and are in a state of long-term exercise, as shown in Table 2.

**TABLE 2:** Descriptive statistics of subjects’ Taijiquan exercise behavior.

Statistic	How long each time	Exercise frequency	Exercise intensity	Years of exercise
M	2.636	3.130	3.267	2.611
SD	0.7827	0.7973	0.5658	1.1802
N	453	453	453	453

M, mean; SD, standard deviation; N, number.

### 3.3 Content validity

To determine the consistency between the TECS-CE and demographic characteristics questionnaire and the research objectives, five experts were invited to conduct the Indexes of Item-Objective Congruence (IOC) test on the scale. After the first round of expert testing, we modified several items below 0.6 and conducted a second expert IOC test. Finally, the average score of the 10 items was 0.8, and that of the other items was 1. The purpose consistency indicators of the questionnaire items satisfied the effectiveness requirements.

### 3.4 Construct validity

#### 3.4.1 Exploratory factor analysis

Based on the recommendations of Dzubian and Shirkey, several indicators were examined to determine the suitability of the TECS-CE inter-item correlation matrix for EFA procedures. Principal component oblique rotation was used to conduct the EFA on the 34 reserved items. The scales of commitment dimensions and commitment determinants were analyzed to test their content and construct validity. As shown in Table 3, the results show that KMO = 0.804 and 0.775 and Bartlett’s test of sphericity *P* < 0.001, indicating that there are common factors among the observed variables that are suitable for factor analysis.

**TABLE 3:** Two dimensions’ and six determinants’ KMO and Bartlett test.

KMO and Bartlett test	KMO sampling suitability quantity	*P*-value
Two dimensions’ KMO and Bartlett test	0.804	<0.001
Six determinants’ KMO and Bartlett test	0.775	<0.001

The principal component method was used for the preliminary EFA. The scale of the commitment dimensions yielded two factors with eigenvalues greater than one, which explained 65.005% of the total variance. Combined with the scree plot, it was found that the questionnaire structure and item distribution were most reasonable when two factors were selected. The two commitment dimensions obtained by EFA are consistent with the original ECS.

The scale of commitment determinants yielded six factors with eigenvalues greater than one, which explained 75.378% of total variance. In the scree plot, there was an inflection point at the sixth factor, indicating that it was most appropriate to extract the six factors. Item PI4 (“I spent some of my own money on exercising Taijiquan”) was deleted due to its low factor loading of 0.333, which falls below the acceptable threshold of 0.40. As shown in Tables 4, 5, the factor loadings, eigenvalues, and variance contribution rates for each item are presented.

**TABLE 4:** Exploratory factor analysis of Taijiquan Exercise Commitment Dimension Scale.

Items	Dimensions	
	Want to	Have to
WC1. I am determined to keep exercising Taijiquan.	0.706	
WC2. I am dedicated to keep exercising Taijiquan.	0.742	
WC3. I am committed to keep exercising Taijiquan.	0.764	
WC4. I am willing to do almost anything to keep exercising Taijiquan.	0.682	
WC5. I want to keep exercising Taijiquan.	0.776	
WC6. It would be hard for me to quit exercising Taijiquan.	0.741	
HC1. I feel obligated to continue exercising Taijiquan.		0.905
HC2. I feel it is necessary for me to continue exercising Taijiquan.		0.862
HC3. I feel exercise Taijiquan is a duty.		0.928
Dimension value	3.808	2.042
Explained variance (%)	42.312	22.692
Cumulative explained variation (%)	42.312	65.005

WC, want to commitment; HC, have to commitment.

**TABLE 5:** Exploratory factor analysis of Taijiquan exercise commitment determination scale.

Items	Determinations					
	IA	IO	PI	SC	Sat	SS
IA2. Compared to exercising Taijiquan, there are other exercises I could do which would be more enjoyable.	0.926					
IA3. Compared to exercising Taijiquan, there are other exercises I could do which would be more worthwhile.	0.912					
IA1. Compared to exercising Taijiquan, there are other exercises I could do which would be more fun.	0.878					
IA4. I would be happier doing other exercises than exercising Taijiquan.	0.867					
IA5. I would like to do exercises other than Taijiquan.	0.788					
IO3. Exercising Taijiquan gives me the opportunity to have a good time.		0.842				
IO1. Exercising Taijiquan gives me the opportunity to feel excited.		0.829				
IO2. Exercising Taijiquan gives me the opportunity to relieve any stress I am feeling.		0.783				
IO4. Exercising Taijiquan gives me the opportunity to be with my friends.		0.778				
IO5. Exercising Taijiquan gives me the opportunity to improve my health and fitness.		0.753				
IO6. Exercising Taijiquan gives me the opportunity to improve my physical skills.		0.693				
PI2. I have invested a lot of energy into exercising Taijiquan.			0.979			
PI3. I have invested a lot of time into exercising Taijiquan.			0.969			
PI1. I have invested a lot of effort into exercising Taijiquan.			0.958			
PI4. I spent some of my own money on exercising Taijiquan.			0.333			
SC4. People are disappointed with me if I quit exercising Taijiquan.				0.917		
SC2. I feel pressure from other people to exercise Taijiquan.				0.912		
SC1. People will think I am a quitter if I stop exercising Taijiquan.				0.794		
SC3. I have to keep exercising Taijiquan to please others.				0.711		
Sat1. All thing considered, exercising Taijiquan is very satisfying.					0.925	
Sat2. Because I exercise Taijiquan, I feel satisfied.					0.922	
Sat3. I find exercising Taijiquan to be very rewarding.					0.918	
SS2. People important to me think it is okay to exercise Taijiquan.						0.906
SS3. People important to me encourage me to exercise Taijiquan.						0.888
SS1. People important to me support my exercising Taijiquan.						0.864
Determination value	5.236	3.413	3.027	2.765	2.322	2.082
Explained variance (%)	20.942	13.652	12.106	11.061	9.289	8.328
Cumulative explained variation (%)	20.942	34.594	46.700	57.761	67.050	

IA, involvement alternatives; IO, involvement opportunities; PI, personal investment; SC, social constraints; Sat, satisfaction; SS, social support.

Finally, the TECS-CE was composed of two dimensions, six factors, and 33 items, which is one item fewer than the original questionnaire. Principal component analysis of the commitment dimensions and determinants was carried out for these 33 items. The two dimensions and six factors explained 65.005 and 75.378% of total variance, respectively. The commitment dimensions consist of two factors and nine items (six for Want to Commit and three for Have to Commit). The determinants of commitment were composed of six factors and 24 items (three for Satisfaction, four for Social Constraints, five for Involvement Alternatives, three for Personal Investment, three for Social Support, and six for Involvement Opportunities).

#### 3.4.2 Confirmatory factor analysis

Using Amos 32.0, the researchers analyzed data from 250 elderly Taijiquan exercisers to verify the commitment dimension and determinant models. Two hypothetical Exercise Commitment Models were examined: Model (M1), a first-order two-factor model with “Want to Commitment” and “Have to Commitment” as related factors, and Model (M2), a second-order three-factor model. The goodness-of-fit results indicated that both hypothetical models demonstrated an acceptable overall fit to the data. For Model MI, the fit indices were χ^2^/df = 1.509, RMSEA = 0.045, RMR = 0.028, AGFI = 0.942, GFI = 0.966, NNFI = 0.993, and CFI = 0.995. For Model MII, the corresponding values were χ^2^/df = 1.371, RMSEA = 0.039, RMR = 0.072, AGFI = 0.882, GFI = 0.903, NNFI = 0.984, and CFI = 0.986. Although Model MII showed lower χ^2^/df and RMSEA values, Model MI demonstrated stronger values for RMR, GFI, AGFI, NNFI, and CFI. Considering the overall pattern of goodness-of-fit indices and the theoretical interpretability of the factor structure, Model MI was retained as the preferred two-factor exercise commitment model. The dimensional structure and model path diagrams are presented in Figures 1, 2.

**FIGURE 1:**
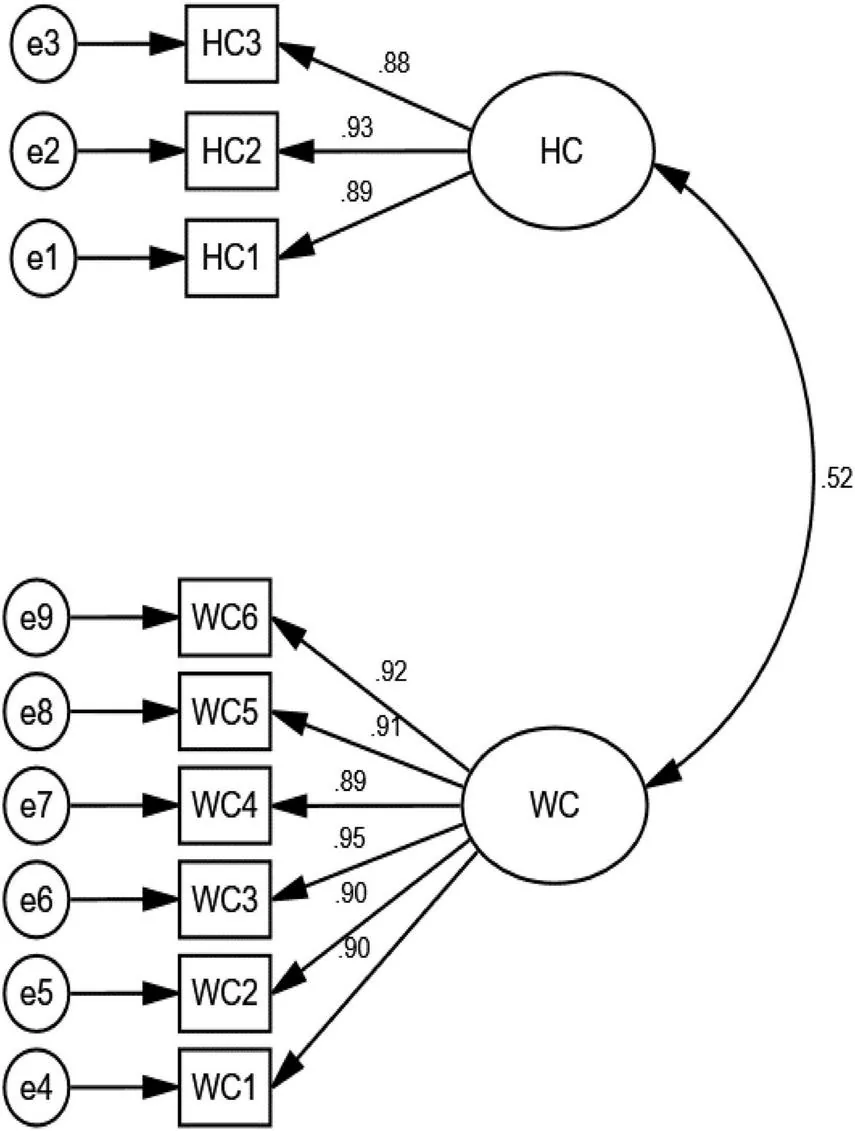
Model diagram of Taijiquan exercise commitment dimension.

**FIGURE 2:**
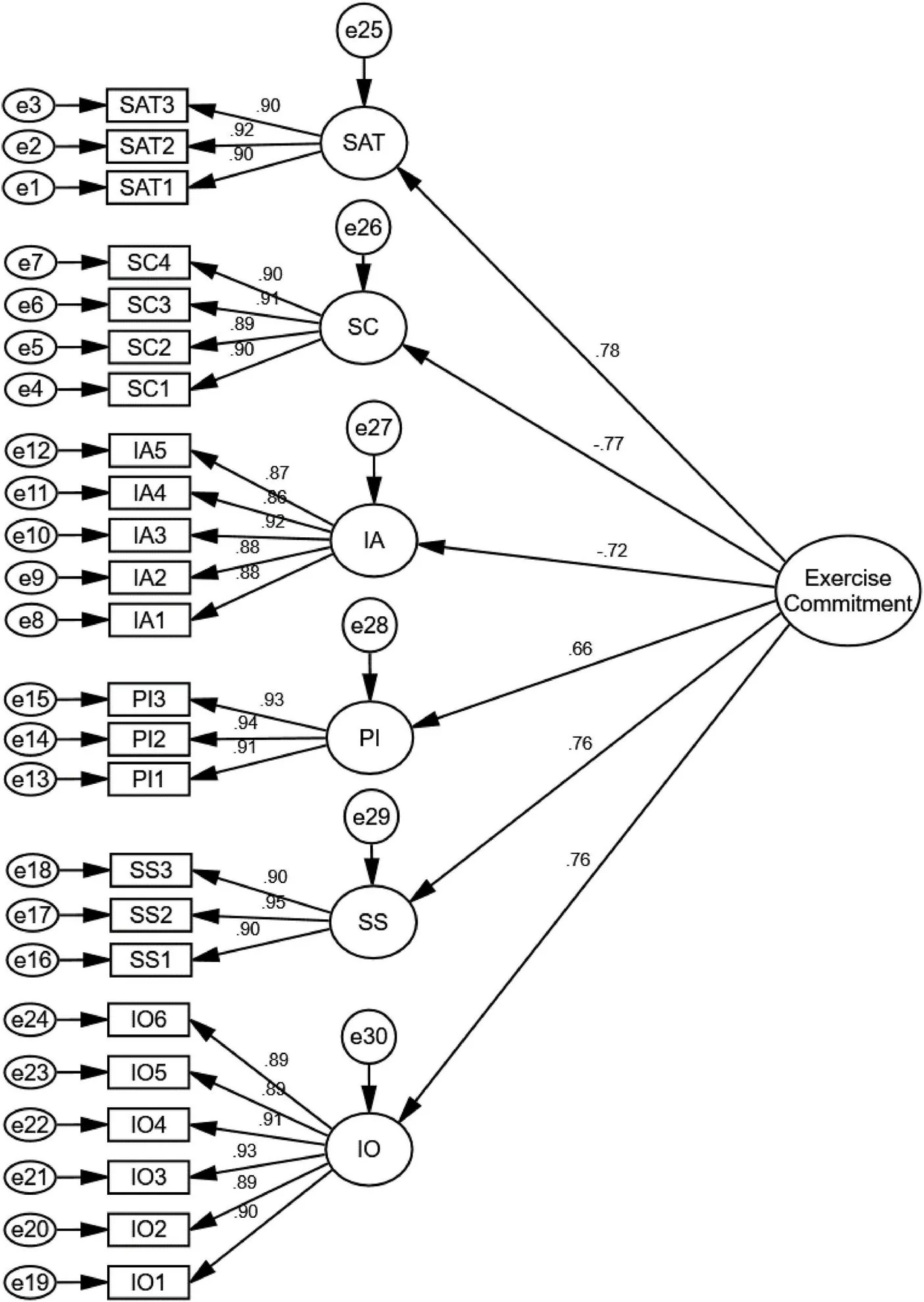
Model diagram of determinants of Taijiquan exercise commitment.

In this study, Exercise Commitment was taken as an Endogenous Latent Variable and six determinant factors as exogenous latent variables. The path diagram of the structural model obtained from the CFA is shown in Table 6. The goodness of fit results of the model is: χ^2^/df = 1.371, *P* = 0.001, RMSEA = 0.039, RMR = 0.072, AGFI = 0.882, GFI = 0.903, NNFI = 0.984, CFI = 0.986. The results showed that each goodness-of-fit index reached an acceptable standard, indicating that the model and data fit well. The six antecedent variables of the model can explain 75.378% (see Table 5) of the variance in exercise commitment (the interpretation rate of cumulative variance after rotation is greater than 50%, indicating that the data can be extracted effectively). This indicates that the six determinant variables specified in the model were significantly associated with the endogenous Taijiquan exercise commitment construct, supporting their proposed roles in the structural model. Correlation analysis was performed for the six factors that determine commitment. The inter-factor correlations ranged from 0.374 to 0.596 in absolute value, indicating moderate associations among the factors. Some factors showed negative correlations, particularly social constraints with satisfaction and social support. These findings suggest that the factors were related but remained conceptually distinguishable, supporting the use of an oblique rotation approach.

**TABLE 6:** Confirmatory analysis results of two hypothetical models.

Model	χ ^2^/df	*p*	RMSEA	RMR	AGFI	GFI	NNFI	CFI
MI	1.509	0.046*	0.045	0.028	0.942	0.966	0.993	0.995
MII	1.371	0.001**	0.039	0.072	0.882	0.903	0.984	0.986

RMSEA, root mean square error of approximation; RMR, root mean square residual; AGFI, adjusted goodness-of-fit index; GFI, goodness-of-fit index; NNFI, non-normed fit index; CFI, comparative fit index. *p < 0.05, **p < 0.01.

Based on the above indicators, the dimensional and determinant models of exercise commitment obtained in this study are reliable.

### 3.5 Convergent and discriminant validity

To further evaluate the construct validity of the TECS-CE, Composite Reliability (CR) and Average Variance Extracted (AVE) were examined. As shown in Table 7, the CR values ranged from 0.91 to 0.94, exceeding the recommended threshold of 0.70, while the AVE values ranged from 0.68 to 0.71, exceeding the recommended criterion of 0.50, indicating satisfactory convergent validity. Furthermore, the square root of the AVE for each construct (0.825–0.843) was greater than the correlation between the constructs (*r* = 0.610), demonstrating satisfactory discriminant validity according to the Fornell–Larcker criterion (Fornell and Larcker, 1981).

**TABLE 7:** Composite reliability, convergent validity, and of the TECS-CE.

Construct	CR	AVE	√AVE
Want to commit (WC)	0.94	0.71	**0.843**
Have to commit (HC)	0.91	0.68	**0.825**
**Construct**	**WC**	**HC**	
Fornell–Larcker Criterion			
Want to commit (WC)	0.843	0.610	
Have to commit (HC)	0.610	0.825	

CR, composite reliability; AVE, average variance extracted. The diagonal elements (bold) represent the square root of the AVE (√AVE), whereas the off-diagonal elements represent the correlation coefficients between constructs. CR values greater than 0.70 and AVE values greater than 0.50 indicate satisfactory convergent validity. Discriminant validity was supported because the square root of the AVE for each construct exceeded the corresponding inter-construct correlation (Fornell and Larcker, 1981).

### 3.6 Criterion-related validity

Relationship between exercise commitment and behavior. Research has shown that maintaining and improving an individual’s commitment to exercise is one of the main factors promoting exercise participation (Wilson et al., 2004). Exercise commitment is more reliable for predicting and explaining exercise behavior through exercise commitment (Chen et al., 2005). Therefore, there is a correlation between exercise commitment and exercise behavior. Within a certain range, the higher the exercise commitment of elderly Taijiquan exercisers, the more eager and determined they were to continue to exercise Taijiquan, the higher the frequency of exercise, the longer the time and intensity of each exercise, and the longer the time they adhered to exercise. In order to test whether the results measured by the TECS-CE were in line with the theoretical assumptions, a correlation analysis between commitment dimensions, determinants, and exercise behavior indicators was carried out. The results are summarized in Table 8. Overall, the scores measured on most subscales were positively correlated with frequency, duration, exercise intensity, and adherence to exercise behavior, while social constraints and participation choices were negatively correlated with exercise behavior.

**TABLE 8:** Correlation analysis between exercise commitment and exercise behavior.

Construct	WC	HC	Sat	SC	IA	PI	SS	IO
Exercise adherence	0.611**	0.610**	0.729**	−0.723**	−0.718**	0.630**	0.731**	0.747**
Exercise frequency	0.395**	0.520**	0.553**	−0.479**	−0.481**	0.536**	0.507**	0.551**
Exercise duration	0.534**	0.507**	0.390**	−0.503**	−0.638**	0.429**	0.565**	0.548**
Exercise intensity	0.486**	0.379**	0.530**	−0.463**	−0.570**	0.453**	0.498**	0.472**

IA, involvement alternatives; IO, involvement opportunities; PI, personal investment; SC, social constraints; Sat, satisfaction; SS, social support. **p < 0.01.

To further explore the criteria related to Taijiquan exercise commitment on exercise behavior, a regression analysis was carried out on the basis of correlation analysis with the dimension of Taijiquan exercise commitment and the determinants of Taijiquan exercise commitment as independent variables and exercise behavior as the dependent variable. Taijiquan exercise behavior was reflected in the total amount of exercise and exercise adherence. Calculation of total physical exercise according to the measurement method of physical exercise. The Taijiquan exercise behavior of the elderly was measured through the intensity, duration, and frequency of physical exercise: Exercise Volume = Exercise duration × exercise frequency × exercise intensity. Thus, the Taijiquan exercise behavior score of each elderly person was calculated, mainly to measure the total amount of participation in Taijiquan exercise for subjects in a week. The results of the regression analysis are presented in Table 9. WC and HC were significantly and positively associated with the total amount of Taijiquan exercise and exercise adherence. Among the commitment determinants, satisfaction, personal investment, social support, and involvement opportunities showed positive associations with both outcomes, whereas social constraints and involvement alternatives showed negative associations. Overall, the commitment dimensions and their determinants were significantly associated with Taijiquan exercise behavior.

**TABLE 9:** Regression analysis of exercise commitment and exercise behavior.

Variables	Total exercise		Exercise adherence	
	***b***	β	***b***	β
Want to commitment	7.982	0.402	0.413	0.412
Have to commitment	7.780	0.388	0.414	0.409
	*R*^2^ = 0.465		*R*^2^ = 0.501	
Satisfaction	2.393	0.119	0.194	0.192
Social constraints	−2.449	−0.119	−0.195	−0.187
Involvement alternatives	−6.225	−0.302	−0.229	−0.220
Personal investment	3.242	0.173	0.123	0.131
Social support	2.772	0.143	0.199	0.203
Involvement opportunities	4.181	0.204	0.253	0.244
	*R*^2^ = 0.684		*R*^2^ = 0.846	

### 3.7 Reliability

To test the reliability of the TECS-CE, Bonett and Wright (2015) pointed out that samples must be as small as 30 to establish reliability, as long as the scale items have strong intercorrelations (Bonett and Wright, 2015). We asked 30 elderly people who could identify personal information to complete the TECS-CE again after 3 weeks to obtain the retest reliability of each dimension. Internal consistency of the scale (Cronbach α Coefficient) and test-retest reliability results are shown in Table 10. Research on the dimensions and determinants of exercise commitment α the co-efficient is between 0.932 and 0.984, which is greater than the recommended value of 0.80, indicating excellent reliability (Carmines and McIver, 1981). They were considered acceptable and met the psychometric requirements. The test-retest reliability ranged from 0.824 to 0.938, indicating a strong correlation and good cross-time stability.

**TABLE 10:** Internal consistency coefficient and test-retest reliability results of TECS-CE.

Construct	WC	HC	Sat	SC	IA	PI	SS	IO
α	0.949	0.942	0.984	0.956	0.932	0.969	0.950	0.941
*R*	0.914	0.896	0.824	0.884	0.884	0.938	0.923	0.918

WC, want to commit; HC, have to commit; IA, involvement alternatives, IO, involvement opportunities, PI, personal investment, SC, social constraints, Sat, satisfaction, SS, social support.

## 4 Discussion and theoretical implications

Taijiquan has been widely recognized as a physical activity that provides substantial health benefits for older adults. However, the participation rate of elderly individuals in Taijiquan exercise in China remains relatively low. Commitment has been defined as “the desire to continue sports participation” (Scanlan et al., 1993). Maintaining and enhancing exercise commitment is considered one of the key factors influencing long-term participation in physical activity (Wilson et al., 2004). To better understand exercise participation among elderly Taijiquan practitioners, this study examined the factors associated with non-participation and withdrawal from Taijiquan exercise. Based on the Exercise Commitment Scale (ECS) developed and validated by Wilson et al., 2004 in, together with the Chinese adaptation of the ECS validated by Qiu et al. among Chinese college students in 2012, the present study developed and validated the Taijiquan Exercise Commitment Scale for Chinese Elderly (TECS-CE). To achieve this objective, five experts were invited to participate in two rounds of Item–Objective Congruence (IOC) evaluation to ensure that the content validity of the TECS-CE met acceptable standards. Subsequently, a questionnaire survey was conducted to collect demographic information, Taijiquan exercise behavior, and exercise commitment characteristics of the participants. Finally, the construct validity, criterion-related validity, and reliability of the TECS-CE were examined through statistical analyses.

The results of the construct validity analysis indicated that the TECS-CE consisted of two dimensions, six factors, and 33 items. The commitment dimension included two factors, namely “want to” and “have to,” comprising nine items. The commitment determinants dimension comprised six factors, including satisfaction, social constraints, involvement alternatives, personal investment, social support, and involvement opportunities, comprising 24 items. One item within the personal investment factor, namely “I spent a lot of money on Taijiquan exercise,” demonstrated a factor loading below the acceptable standard and was therefore removed from the final version of the TECS-CE. This finding differs from both Wilson’s original ECS developed in 2004 and the Chinese ECS validated by Qiu et al. (2012), in which this item demonstrated a positive contribution to exercise commitment and was retained. The exclusion of this item from the TECS-CE may be explained by several contextual factors. First, Taijiquan in China is commonly practiced outdoors and generally does not require specialized equipment, facilities, or substantial financial investment, making participation relatively cost-free (Qiu et al., 2012; Wilson et al., 2004). Second, Chinese government agencies provide various public services and facilities to support national fitness participation among residents. Third, the concept of spending money on sports services may not yet be widely accepted among elderly populations in China.

In addition, social constraints demonstrated a negative effect on the structure of the TECS-CE, which differs from the positive relationship proposed in the Sport Commitment Model (SCM). This finding is consistent with previous studies. For example, Weiss (2020) reported “some inconsistencies in the relationships posited between the determinants and commitment proposed by SCM.” Social constraints have either been unrelated to or negatively correlated with commitment in previous youth sport studies examining SCM (Guo et al., 2016).

These findings may reflect differences in psychological characteristics across age groups participating in physical activity. Elderly individuals may place greater importance on intrinsic feelings, personal enjoyment, and self-satisfaction when engaging in Taijiquan exercise. Many older adults participate in Taijiquan primarily because of personal interest and enjoyment rather than external pressure or obligation. Therefore, excessive or rigid social constraints may not positively influence exercise commitment among elderly participants; instead, such pressures may reduce motivation and create negative feelings toward continued participation.

The factor loadings for each construct ranged from 0.682 to 0.979, indicating acceptable to excellent construct validity. The internal consistency coefficients ranged from 0.923 to 0.984, demonstrating high reliability of the TECS-CE. The goodness-of-fit indices of the measurement model showed satisfactory model fit with the empirical data (χ^2^/df = 1.371, *p* = 0.001, RMSEA = 0.039, RMR = 0.072, AGFI = 0.882, GFI = 0.903, NNFI = 0.984, and CFI = 0.986).

Beyond its role as a measurement instrument, the findings may have implications for the broader application of Taijiquan in healthy aging and rehabilitation contexts. Taijiquan is a low-impact mind–body form of physical activity that may be considered alongside other non-pharmacological approaches for supporting mobility and functional health in older adults. In geriatric and rehabilitation settings, appropriately adapted Taijiquan practice may complement professional care strategies targeting movement control, balance, physical function, and quality of life, particularly when exercise intensity and movement demands are tailored to individual health status and functional capacity (Chu et al., 2022). Accordingly, the TECS-CE may provide complementary information about the psychological and contextual factors associated with older adults’ commitment to such physical activity, while clinical and rehabilitation decisions should remain based on individual assessment and professional judgment.

Furthermore, the TECS-CE demonstrated positive correlations with exercise behavior, except for the dimensions of social constraints and involvement alternatives. The test–retest reliability coefficients ranged from 0.824 to 0.938, indicating strong stability over time. Overall, the psychometric properties of the TECS-CE, including construct validity, criterion-related validity, and reliability, reached acceptable standards. Therefore, the TECS-CE can be used as a valid and reliable instrument for assessing Taijiquan exercise commitment among elderly populations in China.

### 4.1 Implications

China is aging rapidly and is expected to become a super-aging country by 2030. Solving the problems of fitness, medical treatment, entertainment, and exercise in the elderly is an issue that the Chinese government is actively exploring. Taijiquan improves the health of the elderly and is an important form of entertainment, exercise, and medical assistance. Studies that aim to uncover and understand the theoretical underpinnings of traditional activities (Taijiquan) so that appropriately tailored interventions for older adults can be developed (Wilson et al., 2004). This study provides a measurement tool for understanding the elderly’s commitment to Taijiquan exercise and can also provide theoretical support for subsequent research on the elderly’s Taijiquan exercise, as well as theoretical and empirical evidence for the future application of TECS-CE. In addition, the regression analysis in this study showed a positive correlation between exercise behavior and exercise commitment among the elderly. Therefore, this study can also be applied to the intervention of exercise behavior in the elderly, which has a positive significance for the promotion of Taijiquan in the elderly population. For example, sports administrators and instructors can create a positive Taijiquan participation environment in which older adults can achieve their goals, receive recognition, and provide social support. In addition, since satisfaction is an important factor leading to commitment, the elderly’s Taijiquan commitment and behavior toward exercise can also be enhanced if instructors and significant others develop strategies to increase their satisfaction by providing them with positive experiences and a sense of fun during Taijiquan exercise participation.

### 4.2 Study limitations and future research

Several limitations should be considered when interpreting the findings of this study. First, the cross-sectional design limits the ability to establish causal or predictive relationships between exercise commitment and exercise behavior. Although the TECS-CE demonstrated significant associations with reported exercise behavior and satisfactory test–retest reliability, the findings do not demonstrate that commitment causes greater exercise participation or predicts future adherence. Longitudinal studies are warranted to examine changes in commitment over time and determine whether TECS-CE scores predict continued participation or withdrawal over 6- and 12-month follow-up periods.

Second, participants were recruited using convenience sampling from Taijiquan groups in Jiaozuo City, Henan Province, which may limit the generalizability of the findings. The sample may not adequately represent older adults from rural areas, institutional settings, other regions of China, or individuals with different levels of access to community exercise facilities. Regional differences in social environments, availability of public exercise spaces, and community-based Taijiquan programs may influence exercise commitment. Future research should therefore cross-validate the TECS-CE across different geographic and socioeconomic contexts and examine its measurement invariance across urban and rural populations.

Third, all participants had at least 3 years of Taijiquan experience. Consequently, the sample may have consisted primarily of experienced practitioners who were already relatively committed to Taijiquan participation. The findings may therefore not generalize to older adults who are new to Taijiquan or those who are at greater risk of discontinuing exercise. Future studies should include novice, intermediate, and experienced practitioners to examine whether the factor structure and psychometric properties of the TECS-CE remain stable across different stages of participation.

Fourth, exercise behavior was assessed using self-reported measures, including subjective perceptions of exercise intensity. Such measures may be affected by recall bias, individual differences in perception, and social desirability. Therefore, the criterion-related validity findings should be interpreted as evidence of association rather than objective evidence of physical activity or future adherence. Future studies should incorporate objective measures, such as accelerometers, wearable activity trackers, or heart-rate monitoring, to provide stronger evidence for criterion-related validity.

Fifth, the removal of the financial investment item from the personal investment factor may have theoretical implications. The low loading of this item may reflect the relatively low financial demands of community-based Taijiquan practice in China. Thus, personal investment among older Taijiquan practitioners may be more strongly represented by time, physical effort, and personal involvement than by monetary expenditure. Future research should examine whether this conceptualization is applicable across different cultural and socioeconomic contexts.

Finally, although the present study provides evidence supporting the reliability and validity of the TECS-CE, further research is needed to establish how the instrument can practically inform strategies for promoting sustained Taijiquan participation. The eight-factor structure may provide clinicians, physical therapists, sports administrators, instructors, and community health practitioners with a structured framework for assessing different aspects of exercise commitment rather than serving as a diagnostic instrument. In particular, distinguishing between “want to” commitment and “have to” commitment may help practitioners understand whether participation is primarily associated with personal interest and enjoyment or perceived obligation. The determinant factors may further help identify areas that warrant attention, such as satisfaction, social support, involvement opportunities, and social constraints. For example, practitioners may use these domains to inform individualized strategies that strengthen enjoyment, social support, and opportunities for participation while reducing excessive external pressure. In rehabilitation and community health contexts, Taijiquan may also be considered as one low-impact, non-pharmacological physical activity option within broader approaches to maintaining mobility and functional health among older adults, subject to individual health status and professional assessment (Chu et al., 2022).

Previous research has demonstrated the application of the Sport Commitment Model (SCM) to contexts beyond conventional sport participation, including sport injury rehabilitation (Weiss, 2020). In addition, the development of newer exercise commitment measures, such as the multidimensional scale proposed by Scanlan et al. (2016), indicates that exercise commitment can be conceptualized through different theoretical dimensions and determinants. Future research should therefore compare the TECS-CE with both established and newer commitment measures to examine convergent and discriminant validity, theoretical distinctiveness, and relative applicability across different populations and exercise contexts. Importantly, future intervention studies should determine whether assessment of specific TECS-CE domains can be translated into targeted strategies and whether changes in commitment scores are accompanied by improvements in objectively measured exercise participation and long-term adherence.

## 5 Conclusion

This study developed and validated the Taijiquan Exercise Commitment Scale for Chinese Elderly (TECS-CE) as a reliable and valid measure of exercise commitment among older adults practicing Taijiquan. The TECS-CE comprises two dimensions, namely “want to” and “have to” commitment, and six determinants, including satisfaction, social constraints, involvement alternatives, personal investment, social support, and involvement opportunities. The findings provide empirical support for understanding the multidimensional nature of exercise commitment among older Taijiquan practitioners and highlight the potential importance of satisfaction, social support, and other contextual determinants in sustained participation.

The observed association between exercise commitment and exercise behavior suggests that the TECS-CE may be useful for assessing factors associated with Taijiquan participation and for informing the development of future exercise-promotion strategies. In practical settings, sports administrators, instructors, clinicians, physical therapists, and community health practitioners may consider the TECS-CE as one component of a broader assessment of older adults’ exercise experiences and participation context. However, the scale should not be interpreted as a diagnostic or predictive instrument on the basis of the present cross-sectional evidence.

Despite its satisfactory psychometric properties, the findings should be interpreted in light of the study’s sampling characteristics, reliance on self-reported exercise behavior, and cross-sectional design. Future research should employ longitudinal designs, objective measures of physical activity, and samples from diverse geographic and cultural contexts to further examine the generalizability and predictive validity of the TECS-CE. Comparative and cross-cultural validation studies may also clarify its theoretical and practical contribution relative to other exercise commitment measures.

Overall, the TECS-CE provides a culturally relevant instrument for examining exercise commitment among older adults who practice Taijiquan and establishes a foundation for future research on the psychological and contextual factors associated with sustained participation in this traditional form of physical activity.

## Data Availability

The original contributions presented in the study are included in the article/Supplementary material, further inquiries can be directed to the corresponding author.
