# Supplementary material for: Validation of Taijiquan (Tai Chi) Exercise Commitment Scale for Chinese Elderly (TECS-CE)
Source: Front Psychol. 2026 Sep 16;17:1889773. doi: 10.3389/fpsyg.2026.1889773 (PMC13624118; doi:10.3389/fpsyg.2026.1889773)
Supplement: Supplementary file 1 [file Supplementary_file_1.pdf]

Supplementary Material

Appendix A. Final 33-Item Taijiquan Exercise Commitment Scale for Chinese Elderly (TECS-CE).

Dimension	Code	Item	5	4	3	2	1
**Want to Commitment (WC)**	WC1	I am determined to keep exercising Taijiquan.					
	WC2	I am dedicated to keep exercising Taijiquan.					
	WC3	I am committed to keep exercising Taijiquan.					
	WC4	I am willing to do almost anything to keep exercising Taijiquan.					
	WC5	I want to keep exercising Taijiquan.					
	WC6	It would be hard for me to quit exercising Taijiquan.					
**Have to Commitment (HC)**	HC1	I feel obligated to continue exercising Taijiquan.					
	HC2	I feel it is necessary for me to continue exercising Taijiquan.					
	HC3	I feel exercising Taijiquan is a duty.					
**Involvement Alternatives (IA)**	IA1	Compared to exercising Taijiquan, there are other exercises I could do which would be more fun.					
	IA2	Compared to exercising Taijiquan, there are other exercises I could do which would be more enjoyable.					
	IA3	Compared to exercising Taijiquan, there are other exercises I could do which would be more worthwhile.					
	IA4	I would be happier doing other exercises than exercising Taijiquan.					
	IA5	I would like to do exercises other than Taijiquan.					
**Involvement Opportunities (IO)**	IO1	Exercising Taijiquan gives me the opportunity to feel excited.					
	IO2	Exercising Taijiquan gives me the opportunity to relieve any stress I am feeling.					
	IO3	Exercising Taijiquan gives me the opportunity to have a good time.					
	IO4	Exercising Taijiquan gives me the opportunity to be with my friends.					
	IO5	Exercising Taijiquan gives me the opportunity to improve my health and fitness.					
	IO6	Exercising Taijiquan gives me the opportunity to improve my physical skills.					
**Personal Investment (PI)**	PI1	I have invested a lot of effort into exercising Taijiquan.					
	PI2	I have invested a lot of energy into exercising Taijiquan.					
	PI3	I have invested a lot of time into exercising Taijiquan.					
	SC1	People will think I am a quitter if I stop exercising Taijiquan.					

Dimension	Code	Item	5	4	3	2	1
**Social Constraints (SC)**	SC2	I feel pressure from other people to exercise Taijiquan.					
	SC3	I have to keep exercising Taijiquan to please others.					
	SC4	People are disappointed with me if I quit exercising Taijiquan.					
**Satisfaction (Sat)**	Sat1	All things considered, exercising Taijiquan is very satisfying.					
	Sat2	Because I exercise Taijiquan, I feel satisfied.					
	Sat3	I find exercising Taijiquan to be very rewarding.					
**Social Support (SS)**	SS1	People important to me support my exercising Taijiquan.					
	SS2	People important to me think it is okay to exercise Taijiquan.					
	SS3	People important to me encourage me to exercise Taijiquan.					

Note. Response options: 1 = Strongly disagree, 2 = Disagree, 3 = Neither agree nor disagree, 4 = Agree, and 5 = Strongly agree. *IA items are reverse-scored when calculating an overall TECS-CE score because higher scores indicate stronger perceived alternatives to Taijiquan. PI4 (“I spent some of my own money on exercising Taijiquan”) was excluded from the final scale because its factor loading (.333) was below the acceptable threshold. Thus, the final TECS-CE consists of 33 items organized into two commitment dimensions and six commitment determinants, representing eight factors in total.
